# Recent Advances in Histone Methylation in Plant Adaptation to Salinity

**DOI:** 10.3390/plants15131970

**Published:** 2026-06-26

**Authors:** Hammad Hussain, Iqra Noor, Muhammad Adnan Raza, Edvinas Misiukevičius, Ghulam Murtaza, Xinchao Ma, Xiaodong Yang, Hamza Sohail

**Affiliations:** 1 College of Horticulture and Landscape Architecture, Yangzhou University, Yangzhou 225009, China; 2 College of Horticulture and Forestry, Tarim University, Aral 843300, China; 3 Xinjiang Production & Construction Corps Key Laboratory of Facility Agriculture, Tarim University, Aral 843300, China; 4 Department of Agricultural, Food and Nutritional Sciences, University of Alberta, Edmonton, AB T6G2P5, Canada; 5 Institute of Horticulture, Department of Orchard Plant Genetics and Biotechnology, Lithuanian Research Centre for Agriculture and Forestry, Kaunas District, LT-54333 Babtai, Lithuania; 6 Key Laboratory of Biobreeding for Specialty Horticultural Crops of Jiangsu Province, College of Horticulture and Landscape Architecture, Yangzhou University, Yangzhou 225009, China

**Keywords:** epigenetics, histone marks, stress memory, abiotic stress, salt stress

## Abstract

Soil salinization represents one of the most severe abiotic constraints on global agricultural productivity, threatening crop yields and food security across increasingly large areas of cultivated land. Among the molecular mechanisms underlying plant physiological adaptation to salinity, histone methylation has emerged as a central epigenetic regulatory layer governing salt-responsive transcriptional reprogramming through the coordinated and opposing actions of histone methyltransferases, demethylases, and reader proteins at specific chromatin loci. Recent advances reveal how dynamic changes in activating marks, principally H3K4me3 and H3K36me3, and repressive marks, H3K9me2 and H3K27me3, orchestrate the activation of stress-responsive gene networks and the silencing of growth-incompatible programs under salt stress. How these modifications establish and sustain stress memory across somatic and transgenerational timescales is discussed. Recent technological advances, including single-cell epigenomics, CUT&RUN, CUT&Tag, and spatial transcriptomics, are assessed as future research priorities. The application of CRISPR/dCas9-based epigenome editing and epigenetic breeding strategies for improving crop salt tolerance is further explored.

## 1. Introduction

**Figure 1:**
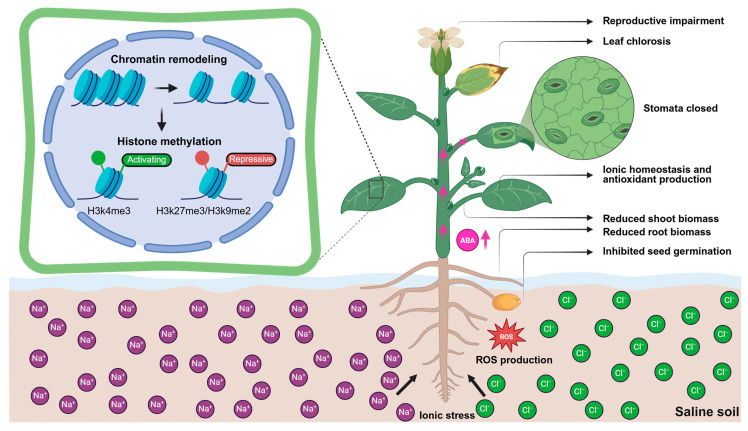
Epigenetic and physiological responses of plants to salinity stress. Salinity stress triggers a dual-level response in plants encompassing epigenetic reprogramming and whole-plant physiological adaptation. Na^+^ and Cl^−^ ions accumulate in saline soil, imposing ionic stress and stimulating reactive oxygen species (ROS) production at the root interface. These signals elevate abscisic acid (ABA) levels, mediating downstream stress responses. At the cellular level, salt stress initiates chromatin remodeling, transitioning nucleosomes from a condensed to a relaxed state and enabling differential histone methylation: H3K4me3 acts as an activating mark promoting stress-responsive gene expression, while H3K27me3 and H3K9me2 function as repressive marks silencing growth-related genes. At the whole-plant level, salinity induces reproductive impairment, leaf chlorosis, stomatal closure, reduced shoot and root biomass, inhibited seed germination, ionic homeostasis disruption, and antioxidant enzyme induction.

Soil salinity, a major abiotic stress impacting global agriculture, affects over 20% of cultivated land and roughly half of all irrigated regions [1,2,3]. Plants, like other sessile organisms, are sensitive to salinity stress, and it is critical to recognize that salinity does not act as a singular stressor but rather as a complex syndrome characterized by a dual-phasic response: an initial osmotic phase followed by a more protracted ionic phase [4,5]. Osmotic stress reduces water uptake, while ionic stress enhances the accumulation of Na^+^ and Cl^+^ ions in plant tissues [6]. These phases collectively trigger a cascade of morphological, physiological, and biochemical adjustments that define a plant’s survival and productivity [7]. Morphologically, salinity inhibits germination, reduces shoot and root biomass, alters root-to-shoot partitioning, and accelerates leaf chlorosis and senescence (Figure 1) [8,9,10,11,12,13,14]. Physiologically, the osmotic component imposes a “physiological drought” that drives stomatal closure, restricts CO_2_ assimilation, and damages chloroplast ultrastructure, while ionic toxicity disrupts K^+^/Na^+^ homeostasis and activates NADPH-oxidase–dependent reactive oxygen species (ROS) production [12,15,16,17]. Biochemically, excess ROS causes lipid peroxidation and membrane injury, prompting plants to deploy enzymatic antioxidants (SOD, CAT, POD), to accumulate compatible solutes such as proline, glycine betaine, and soluble sugars, and to engage phytohormone networks, most prominently ABA-mediated signaling [12,18,19,20]. Each of these adaptive outputs depends on rapid and coordinated transcriptional reprogramming of stress-responsive gene networks, which is achievable only through dynamic remodeling of chromatin accessibility at the relevant loci. It is at this regulatory interface that epigenetic mechanisms, and histone methylation in particular, have emerged as decisive determinants of plant salt tolerance, motivating the present review.

Epigenetics broadly refers to chromatin-associated regulatory mechanisms, including DNA methylation and histone modifications, that modulate gene expression without altering the underlying nucleotide sequence. In some cases, these epigenetic states can be transmitted through cell divisions or across generations, although such heritability is context-dependent and not universal. In the context of salinity stress, these mechanisms provide plants with a sophisticated regulatory system to fine-tune their molecular and physiological responses to environmental cues. Unlike permanent genetic mutations, epigenetic changes allow for phenotypic plasticity, offering a flexible and reversible way to coordinate salt stress responses with overall growth and development. These modifications, which include DNA methylation, histone tail alterations, and the activity of non-coding RNAs, fundamentally restructure chromatin architecture, thereby regulating its accessibility to the transcriptional machinery [21].

**Figure 2:**
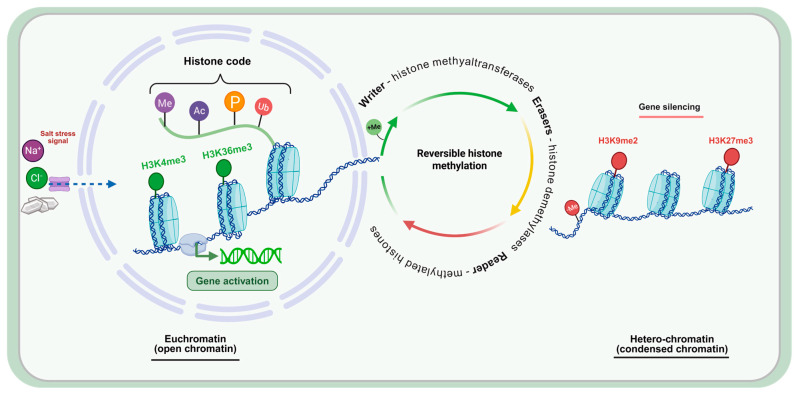
Histone methylation dynamics and chromatin states in plant responses to salinity stress. Salt stress signals, conveyed by Na^+^ and Cl^−^ ions, enter the plant cell and trigger epigenetic reprogramming through differential histone methylation. Within the nucleus, histone N-terminal tails carry post-translational modifications collectively termed the “histone code,” including methylation (Me), acetylation (Ac), phosphorylation (P), and ubiquitination (Ub). Under salt stress, activating marks H3K4me3 and H3K36me3 accumulate at stress-responsive loci, promoting open chromatin (euchromatin) conformation and gene activation. Histone methylation is maintained through a reversible cycle mediated by three classes of regulatory proteins: writers (histone methyltransferases, HMTs) that deposit methyl groups (+Me), erasers (histone demethylases, HDMs) that remove methyl groups (−Me), and readers (methylated histones) that interpret the methylation state to coordinate transcriptional outcomes. In contrast, repressive marks H3K9me2 and H3K27me3 drive chromatin compaction into heterochromatin (condensed chromatin), resulting in gene silencing. The dynamic interplay between these opposing chromatin states enables precise transcriptional reprogramming of salt-responsive gene networks.

To cope with saline environmental conditions, plants have developed complex molecular networks to convert external environmental signals into internal adaptive responses. A critical layer of this adaptive response is the epigenetic regulation of gene expression through reversible, heritable changes to gene activity without altering the underlying sequence of DNA [22,23]. A central regulatory layer in epigenetic control is histone post-translational modification (PTM), operating principally through the N-terminal tails of histone proteins that interact with DNA to form nucleosomes [24]. These modifications are collectively called the “histone code” and define the chromatin landscape’s accessibility to transcriptional regulation. The most important types of PTMs to histones include acetylation, methylation, phosphorylation, and ubiquitination (Figure 2) [25,26]. Acetylation of histones is associated with neutralizing lysine positive charges and creating transcriptionally active (open) chromatin, while other modifications such as phosphorylation and ubiquitination play important roles in signaling and stress response [27,28].

Among histone PTMs, methylation is one of the most complex and versatile forms of epigenetic regulation of gene expression occurring on K and R residues of histones. Methylated histones may have different functional outcomes depending on where the methylation occurs and how many methyl groups were added to the residue (i.e., mono-, di-, or trimethylation) [29]. In general, histone methylation is maintained and regulated by the balanced dynamic processes of “writing” (histone methyltransferases), “erasing” (histone demethylases), and interpreting “reading” (methylated histones) of the respective PTM (Figure 2). Methylation of H3 at K4 and K36 is generally associated with gene activation and euchromatin, while methylation of H3 at K9 and K27 is generally associated with gene silencing and heterochromatin (Figure 2) [30]. By using the multifaceted types of methylation, plants can precisely modify their transcriptomes in response to salt stress. Current evidence suggests that in addition to regulating the immediate response to salinity stress, histone methylation also regulates “memory of stress,” which allows plants to respond more effectively to intermittent salinity stress after maintaining a specific chromatin configuration [31].

Despite these findings, how the histone methylation marks collectively orchestrate plant physiological adaptation to salt stress remains limited. Therefore, this review systematically examines the recent advancements of histone methylation under salt stress, from the fundamental enzymatic machinery governing these epigenetic marks to their functional consequences in mediating salinity responses in plants. Finally, we will evaluate the potential to utilize these epigenetic regulatory pathways to enhance crop productivity and resilience to salinity stress.

## 2. Mechanism of Histone Methylation Under Salt Stress in Plants

Among the diverse PTMs that remodel chromatin architecture under environmental stress, histone methylation has emerged as a central regulatory mechanism governing salt-responsive transcriptional reprogramming in plants [32,33,34,35]. In recent years, researchers have concentrated on elucidating the molecular signal transduction pathways and transcriptional cascades activated under salt stress [36,37,38]. Recent studies have strengthened the literature demonstrating the essential role of epigenetic regulation in altering the expression of salt-responsive genes, particularly through histone methylation [39,40,41]. Salinity elevates the prevalence of H3K4me3 marks at stress-responsive genes, including *RD29A*, *RD29B*, *COR15A*, and *COR15B*, promoting their transcription [42]. In other species such as soybean *Glycine max* (L.) Merr., salt stress can also reduce H3K9me2 levels at certain stress-related genes [43]. Moreover, more than 4000 genes in *Arabidopsis thaliana* (L.) Heynh. plants have exhibited alterations in their H3K4me3 levels in response to salt stress, demonstrating a strong correlation with other pathways linked to abiotic stressors [42]. In *Glycine max* (L.) Merr., similar epigenetic reprogramming has been observed during salt stress, which induces H3K4me3 and H3K4me2 methylation of several genes, facilitating ion homeostasis and cell wall remodeling [44]. In rice (*Oryza sativa* L.), H3K4me3 and H3K27me3 patterns at the *OsBZ8* locus differentiate salt-tolerant and salt-sensitive cultivars [45]. Moreover, *RSM1*, an ABA-responsive transcription factor, was identified as being regulated by the balance between H3K4me3 and H3K27me3 during salt stress in castor bean (*Ricinus communis* L.) [46]. All of these findings further underline the pivotal role of histone methylation in response to salinity.

**Figure 3:**
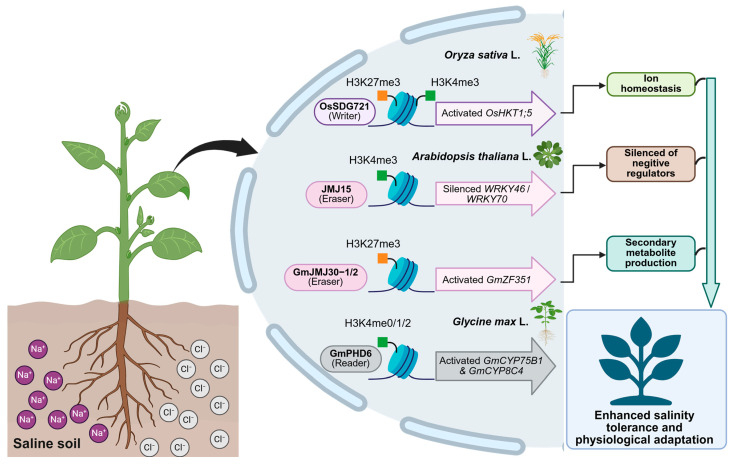
Examples of histone methylation-mediated transcriptional reprogramming under salinity stress in plants. Salinity stress imposes ionic and osmotic stress through the accumulation of Na^+^ and Cl^−^ ions in the rhizosphere, triggering epigenetic reprogramming within the plant cell nucleus. Four mechanistic axes of histone methylation regulation are illustrated. The SET DOMAIN GROUP protein OsSDG721 functions as a histone methyltransferase (writer) depositing H3K4me3 at the *OsHKT1;5* locus in *Oryza sativa*, replacing the repressive H3K27me3 mark and activating Na^+^ transporter expression to restore ion homeostasis. The H3K4me3 demethylase JMJ15 functions as an eraser at the *WRKY46* and *WRKY70* loci in *Arabidopsis thaliana*, removing activating H3K4me3 marks and silencing these negative regulators of salt tolerance. The demethylases GmJMJ30-1/2 erase repressive H3K27me3 marks at the *GmZF351* locus in *Glycine max*, activating stress resistance and secondary metabolite production pathways. The PHD-domain reader protein GmPHD6 recognizes H3K4me0/1/2 methylation states at the *GmCYP75B1* and *GmCYP82C4* loci, activating secondary metabolite biosynthesis. Collectively, these histone methylation-mediated transcriptional outputs converge on enhanced salinity tolerance and physiological adaptation.

Enzymes that facilitate histone methylation and demethylation are crucial in plant response to salinity. *HKT1* genes encode members of the *High-affinity Potassium Transporter* (*HKT*) family, which are integral membrane proteins that function as critical monovalent cation transporters in plants. Specifically, the *HKT1* subfamily (also known as Group 1 *HKTs*) predominantly acts as Na^+^ uniporters that are essential for protecting plants from salinity by maintaining low sodium levels in sensitive shoot tissues. These transporters are primarily localized in root xylem parenchyma cells, where they retrieve Na^+^ from the xylem sap to prevent its translocation from the roots to the leaves [47]. The *HKT1* is a critical component of Na^+^ transportation in plants under salinity that also interacts with salt-overly sensitive pathways [48]. The *HKT1* gene’s body was shown to have the H3K27me3 mark, and the activation of the gene is positively correlated with the removal of the repressive mark [49,50]. The regulation of *OsHKT1;5* expression in *Oryza sativa* L. has been proven to be mediated through regulation of the H3K4me3 level via the SET DOMAIN GROUP protein SDG721 (Figure 3) [51]. Furthermore, histone demethylases aid in plant adaptive responses to salinity stress. JMJ (Jumonji) proteins represent a specialized family of histone demethylases that act as vital epigenetic regulators within the molecular networks of plants. According to the sources, these proteins are integral to chromatin remodeling processes, which facilitate the conversion of chromatin from a transcriptionally inactive state to an active one, thereby allowing for the precise regulation of stress-responsive genes. In transcriptional studies of crops like guar, members of the *JMJ* family have been identified as differentially expressed genes (DEGs) in response to salt stress, highlighting their significant role in the plant’s adaptive transcriptional reprogramming. By modifying the methylation status of histones, *JMJ* demethylases serve as key players in maintaining cellular integrity and orchestrating the complex biological pathways required for salinity tolerance [52]. For example, the *Arabidopsis thaliana* (L.) Heynh. ecotype Columbia (Col-0) H3K4me3 demethylase *JMJ15* has been shown to mediate salt tolerance, and gain-of-function mutants of *jmj15* have improved performance under salt stress conditions [53]. *JMJ15* interacts with and targets negative regulators *WRKY46* and *WRKY70* by removing the H3K4me3 mark from their histone tails, thereby repressing their transcription (Figure 3) [42]. Lastly, many other members of the *JMJ* family of genes have been induced via salt stress treatment in other agronomically important crops such as rice (*Oryza sativa* L.) and cotton (*Gossypium hirsutum* L.), and the overexpression of members of the *JMJ* gene family in yeast systems is associated with improved salt tolerance [54,55]. The CCCH zinc finger protein GmZF351, which participates in oil deposition and various stress resistance mechanisms in *Glycine max* L., has been shown to have its expression levels modulated by the two *GmJMJ30-1/2* demethylases through lowering levels of the histone modification H3K27me3. The overexpression of *GmJMJ30-1/2* genes increases salt tolerance in transgenic hairy roots (Figure 3) [56]. In addition, GmPHD6 is shown to be a “reader” of histone modifications by interacting with and recognizing the different methylation states of histone H3K4 (H3K4me0/1/2) and regulating the transcription of salt-responsive genes such as *GmCYP75B1* and *GmCYP82C4* (Figure 3) [57]. Despite these mechanistic advances, the relationship between stress-induced histone methylation changes and transcriptional reprogramming is more complex than a simple cause-and-effect model suggests. Several lines of evidence challenge the simplistic view that stress-induced changes in histone methylation directly and universally drive transcriptional reprogramming. A genome-wide analysis revealed that after a short salt priming treatment in *Arabidopsis thaliana* (L.) Heynh. (Col-0) roots, most genes undergoing changes in H3K4me3 or H3K27me3 were not differentially expressed, and only 50% of genes with overlapping changes in a histone mark and gene expression followed the expected correlation [49]. This observation extends to crop species: in peas (*Pisum sativum* L.), a comprehensive ChIP-seq and RNA-seq analysis under salt stress demonstrated that changes in histone modifications may not directly cause variations in gene expression and that H3K27me3-marked genes were predominantly linked to medium levels of expression deviating from prior research that typically associates this mark with lowly expressed genes [58]. Moreover, in gain-of-function mutants of the H3K4 demethylase *JMJ15*, most differentially expressed genes were downregulated under control conditions, yet selected salt-responsive genes (*RD29A*, *RD29B*, *RD22*, *COR15A*, *COR47*, *P5CS1*, and *P5CS2*) were actually hyper-induced after salt treatment compared to the wild type, suggesting they may not be direct targets of *JMJ15* [53]. Perhaps most counterintuitively, H3K27me3 has been shown to accumulate on salt-induced genes during salt treatment in *Arabidopsis thaliana* (L.) Heynh. (Col-0), functioning as a regulatory brake alongside histone deacetylation to attenuate transcriptional over-activation of stress-responsive genes [59]. Concerning stress memory, while H3K4me3 retention at the *P5CS1* locus has been mechanistically linked to enhanced transcriptional responses upon repeated salt exposure [60], a mechanistic link between priming-induced changes in H3K27me3 and altered transcriptional profiles upon stress re-occurrence after longer recovery periods remains unproven [49]. Together, these findings indicate that transcriptional stress responses can occur without accompanying changes in histone modifications, and conversely, many genes display altered histone marks without a corresponding change in transcription [49,53]. Beyond the complexity of mark transcription correlations, the upstream signals that connect ionic stress perception to histone methylation machinery remain poorly defined. Evidence for direct crosstalk between Ca^2+^ signaling and histone methylation in plants remains limited to a single characterized axis. In *Arabidopsis thaliana* (L.) Heynh. (Col-0), the arginine methyltransferase CAU1/PRMT5/SKB1 epigenetically silences the extracellular Ca^2+^ sensor gene *CAS* via H4R3sme2 deposition, a repression relieved when elevated Ca^2+^ reduces CAU1 protein abundance and demethylates the *CAS* locus to trigger stomatal closure [61]. Whether analogous Ca^2+^-dependent regulation of chromatin methylation states operates at salt-responsive loci under ionic stress remains unresolved, underscoring the need for targeted investigation of this signaling epigenome interface. Table 1 provides a consolidated overview of histone methylation marks, their targeted genes, and mechanistic outcomes under salt stress across diverse plant species.

## 3. Role of Histone Methylation in Salt Stress Memory

Epigenetic memory in plants involves stable yet reversible chemical modifications such as histone modifications, DNA methylation, and chromatin remodeling that allow a plant to “remember” previous environmental stresses and adjust its future transcriptional responses. In the context of histone modifications, specific regulators like the *JMJ* family of histone demethylases and SNF2-family chromatin remodeling factors play vital roles in shifting chromatin from a transcriptionally inactive state to an active one to execute adaptive stress responses [73,74]. The duration of epigenetic memory in plants depends on whether plants remember their response to stress or not and may last for days, weeks, or years after the exposure to the trigger, depending on the plant species and the environmental setting where it grows [75]. Somatic stress implies maintenance of chromatin modification throughout the lifespan of a plant. The latter lasts for several days or weeks after the first trigger’s action ends [76]. For example, in *Arabidopsis thaliana* (L.) Heynh. (Col-0), salt-induced transcriptional memory and acquired stress tolerance have been shown to last for at least 10 days when the plant remains “ready to respond” [49,60]. In some monocots such as *Oryza sativa* L., somatic stress memory has been demonstrated to be as long as 45 days. The most amazing thing about stress-induced somatic memory is that its effects may be transferred to the dormant seed state, allowing offspring of these plants to acquire salinity resistance already upon germination, lasting for 2 years after the triggering event [77].

Histone methylation stands out as a central pillar of plant stress memory, a phenomenon where prior exposure to salinity primes the plant for a more robust or rapid response upon re-exposure [78]. Unlike more transient modifications like acetylation, histone methylation, particularly at H3K4 and H3K27, is generally associated with longer-term regulatory processes and transcriptional memory that can persist across cell divisions [7,58]. These modifications act as a “transcriptional bookmark,” keeping genes in a poised or “ready” state even after the initial stress has subsided [79,80,81]. Proline is a critical compatible solute and osmoprotectant that plants accumulate in the cytoplasm to maintain cell turgor without interfering with normal metabolic activities. It plays a paramount role in osmotic regulation and the stabilization of cellular structures, including proteins and membranes, under the physiological drought conditions induced by salinity. Beyond its function as an osmolyte, proline serves as a molecular chaperone, a source of carbon and nitrogen, and a potent scavenger of ROS to mitigate oxidative damage. Under severe salinity stress, endogenous proline levels can remarkably increase compared to normal conditions. Furthermore, proline is involved in cellular signaling and the maintenance of cytosolic pH, and its rapid breakdown provides essential reductants for ATP production to support recovery from salt-induced injury. In many crop species, such as wheat (*Triticum aestivum* L.) and *Glycine max* (L.) Merr., higher proline accumulation is frequently used as a biochemical indicator of enhanced salt tolerance [82].

**Figure 4:**
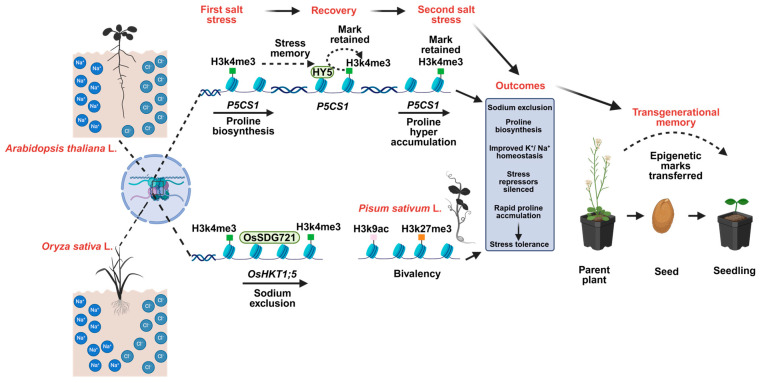
Examples of Histone methylation as a mechanism of salt stress memory in plants. Salt stress perception triggers epigenetic reprogramming within the plant cell nucleus, establishing histone methylation-based transcriptional memory that primes faster and stronger responses upon repeated salinity exposure. At the *P5CS1* locus in *Arabidopsis thaliana*, the first salt stress induces H3K4me3 deposition, activating proline biosynthesis. During recovery, the H3K4me3 mark is retained as a transcriptional bookmark maintained by the transcription factor HY5 even as *P5CS1* mRNA returns to basal levels. Upon second salt stress, the pre-existing H3K4me3 enables accelerated transcriptional machinery recruitment and proline hyper-accumulation. In *Oryza sativa*, the H3K4 methyltransferase OsSDG721 deposits H3K4me3 at the *OsHKT1;5* locus, activating sodium exclusion and maintaining K^+^/Na^+^ homeostasis under recurring stress. A bivalent chromatin state characterized by the coexistence of the activating mark H3K9ac and the repressive mark H3K27me3 at stress-signaling gene promoters in *Pisum sativum* maintains genes in a repressed-but-ready configuration, permitting rapid metabolic reprogramming upon saline re-exposure. Collectively, these histone methylation-mediated memory mechanisms converge on enhanced stress tolerance through sodium exclusion, proline biosynthesis, improved K^+^/Na^+^ homeostasis, and silencing of stress repressors. Stress-induced epigenetic marks can additionally be transmitted to offspring via the seed, conferring salinity resistance upon germination and persisting for up to two years.

A key example of this transcriptional memory occurs at the *P5CS1* locus, which is related to proline biosynthesis. Previous exposure to salt induces high levels of the active mark H3K4me3 at the *Δ1-pyrroline-5-carboxylate synthetase 1 (P5CS1)* locus. During recovery, while *P5CS1* mRNA levels return to basal states, the H3K4me3 mark is retained, facilitating a much faster recruitment of transcriptional machinery and hyper-accumulation of proline during a second salt challenge (Figure 4). This memory maintenance is integrated with light signaling, specifically requiring the transcription factor HY5 to sustain H3K4me3 levels during the recovery phase [60,83]. In *Oryza sativa* L., salt stress induces the H3K4 methyltransferase OsSDG721, which deposits the active H3K4me3 mark at the promoter and coding regions of *OsHKT1;5*. This upregulation is vital for sodium exclusion and maintaining K^+^/Na^+^ homeostasis, with the epigenetic state of this locus determining the plant’s ability to “remember” and maintain transport efficiency under recurring stress [40,51]. A unique bivalent chromatin state (H3K9ac-H3K27me3) has been discovered in *Pisum sativum* L. plants, where activating and repressive marks coexist at the promoters of stress-signaling genes. This state is thought to keep genes in a “repressed but ready” condition, allowing for rapid metabolic reprogramming when saline conditions return [58,84] (Figure 4). In *Arabidopsis thaliana* (L.) Heynh. In Col-0, the JmjC-domain demethylase *JMJ15* acts as an “eraser” that removes H3K4me2/3 marks from the promoters of negative stress regulators like *WRKY46* and *WRKY70*. By suppressing these negative regulators through H3K4 demethylation, the plant maintains a higher hierarchical state of salt defense [28,42,85].

## 4. Histone Methylation as an Epigenetic Strategy to Improve Crop Productivity and Salinity Stress Tolerance

The increasing salinity of agricultural lands highlights the urgent need to improve salt tolerance in crops, a critical factor for ensuring food security. Understanding histone methylation regulatory networks is therefore critical for coping with the negative impact of salinity stress on crop productivity and, ultimately, for the rational design of strategies to improve plant performance. The value of histone methylation in this context lies in its capacity to dynamically regulate stress-responsive transcriptional networks while preserving developmental plasticity. Unlike permanent genetic alterations, histone methylation marks function as reversible epigenetic switches that orchestrate ion homeostasis, ROS detoxification, hormonal signaling, and osmotic adjustment, making histone methyltransferases and demethylases attractive targets for developing salinity-resilient crops [28,78]. Functional evidence across multiple species, from model plants to major crops, increasingly supports this translational potential. In *Arabidopsis thaliana* (L.) Heynh. In Col-0, several histone methylation modules have been mechanistically characterized as direct regulators of salinity tolerance. The CDK8–AHL10–SUVH2/9 module regulates salt tolerance via H3K9me2 deposition. Salt stress activates CDK8, which phosphorylates and degrades AHL10, reducing SUVH2/9 recruitment and H3K9me2 at salt-responsive gene promoters, thereby relieving transcriptional repression [86]. Additionally, the E2 ubiquitin carrier proteins UBC1 and UBC2 enhance salt tolerance by promoting H2Bub1-dependent H3K4me3 deposition at *MYB42* and *MPK4* loci. This epigenetic activation upregulates *MYB42*-driven *SOS2* expression, thereby activating the SOS pathway to maintain Na^+^/K^+^ homeostasis [87]. Similarly, salt stress drives chromatin remodeling at *PP2C* gene loci, characterized by nucleosome eviction, increased H3 acetylation and H3K4me3, and transcription factor switching from *AtMYB44* to *ABF3*, collectively shifting chromatin from a repressive to a transcriptionally active state. Concurrently, BRAHMA ATPase independently contributes to transcriptional repression at these loci [88].

Building on these mechanistic foundations, functionally parallel regulatory axes have been documented across economically important crop species, reinforcing the broader applicability of histone methylation pathways for crop improvement. In *Oryza sativa* L., the *IDS1*–*TPR1*–*HDA1* complex represses *LEA1* and *SOS1* expression through histone H3 deacetylation, thereby compromising salt tolerance [89], while ARGONAUTE2 (AGO2) simultaneously enhanced grain length and salt tolerance by modulating histone methylation at the BIG GRAIN3 (BG3) locus to promote its transcriptional activation [90]. In *Glycine max* (L.) Merr., genome-wide ChIP-seq analysis revealed that dynamic changes in H3K27me3, mediated by specific histone methyltransferases and demethylases, act as a molecular switch regulating the activation or silencing of salt-responsive genes in roots [64]. In maize (*Zea mays* L.), the H3K36 methyltransferase SDG102 positively regulated salt tolerance by governing genes involved in ion transport, antioxidant defense, and hormone signaling, while the JmjC-domain demethylase *ZmJMJ703* was shown to be equally indispensable, with its loss impairing amino acid metabolism, protein trafficking, and secondary metabolite biosynthesis under saline conditions [70,91]. In *Gossypium hirsutum* L., salt stress reduces H3K27me3 deposition at the *GhNCED3* and *GhTIFY9* loci, relieving transcriptional repression of these genes and thereby enhancing salt tolerance through modulation of Na^+^/K^+^ homeostasis and ROS scavenging [69]. In alfalfa (*Medicago sativa* L.), salt stress promotes DNA demethylation and enrichment of H3K9ac and H3K4me3 at the *MsMYB4* promoter, transcriptionally activating this R2R3-MYB factor to confer enhanced salt tolerance [63]. Across these diverse crop species, a convergent pattern emerges: salinity stress consistently shifts chromatin from a repressive to a transcriptionally permissive state at key tolerance loci, primarily through coordinated modulation of H3K4me3, H3K27me3, and H3K9me2 marks, a mechanistic logic that offers a compelling rationale for deliberately engineering these chromatin states to improve crop resilience.

## 5. Future Prospects

The growing catalogue of research pertaining to histone methylation with respect to plant responses to salinity stress has produced numerous mechanistic insights; however, there are still large gaps in the translation of this information into applicable agricultural practices. Accordingly, future research should shift from descriptive studies to integrative, high-resolution, and application-focused studies that facilitate bridging the gap between epigenetic regulation of plants and improvement of crops.

One of the highest priorities is the generation of high-resolution epigenomic maps under conditions of salinity stress. Current epigenomic research has demonstrated global changes in histone marks such as H3K4me3, H3K27me3, and H3K9me2 during salinity stress; however, most of the studies have sampled at least the majority of their tissue from bulk tissue samples instead of monoclonal plant cells. Since different plant cell populations, including root epidermal cells, xylem parenchyma, and guard cells, do not respond to salinity via the same epigenetic mechanisms, the adoption of single-cell epigenomics technologies such as single-cell ATAC-seq and single-cell ChIP-seq will be essential to map chromatin states at cellular resolution. CUT&RUN and CUT&Tag have emerged as low-input alternatives to conventional ChIP-seq that require substantially fewer cells and less starting material, making them particularly advantageous for profiling histone methylation marks in plant tissues where chromatin yield is inherently limiting; however, their adoption in plant epigenomics research remains limited and their application to salinity stress contexts is largely unexplored. Spatial transcriptomics extends single-cell resolution genomics by retaining the spatial coordinates of cells within intact tissue architecture, enabling the simultaneous mapping of transcriptional and chromatin states across tissue domains, a capability that would allow dissection of cell-type-specific histone methylation responses to salinity at the level of individual tissue layers such as root epidermis and endodermis. Multi-omics platforms capable of simultaneously profiling multiple histone modifications within the same cell would further resolve the combinatorial relationships between activating and repressive marks such as H3K4me3 and H3K27me3 and their coordinated dynamics during transitions between stress chromatin states. Long-read sequencing platforms, while primarily applied to DNA methylation and chromatin accessibility rather than histone modifications directly, hold future potential for resolving epigenetic states at repetitive genomic regions that are inaccessible to short-read ChIP-seq.

Collectively, these methodological advances must be coupled with the integration of multi-omics datasets, including transcriptomics, proteomics, and metabolomics. Since histone methylation does not operate in isolation but acts in conjunction with transcription factors, non-coding RNAs, and metabolic pathways, systems-biology approaches that integrate these datasets will be critical for building comprehensive regulatory networks that define how plants respond to salt stress. Of particular importance will be the incorporation of time-course experimental designs, which will assist in distinguishing regulatory events that are causally upstream of transcriptional responses from those that are merely downstream consequences.

A further limitation of the current literature is its heavy reliance on the model plant *Arabidopsis thaliana*. While mechanistic insights from model species provide an essential foundational knowledge base, their direct translation to crop species remains uncertain due to fundamental differences in genomic organisation, epigenetic landscape complexity, and stress physiology. Future epigenomic studies must therefore prioritise agronomically important species such as rice, wheat, maize, and soybean, and comparative epigenomic analyses between model and crop species will be essential to distinguish conserved from species-specific regulatory mechanisms. A conspicuous gap in the current literature is the near-complete absence of primary studies characterizing histone methylation dynamics in halophytic species and economically important horticultural crops such as tomato and grapevine under salinity stress; addressing this gap through targeted ChIP-seq and multi-omics approaches in these systems represents a priority for translating mechanistic insights from model species into salt-tolerant crop improvement. Alongside this, the role of non-coding RNAs in directing histone methylation in crop species under salt stress warrants greater attention. Small RNAs and long non-coding RNAs have been shown to recruit chromatin-modifying complexes to specific genomic loci, thereby modulating histone methylation patterns, yet their contribution to salt-stress epigenomic reprogramming in crops remains poorly characterized.

The rise of CRISPR/dCas9-based epigenome editing technologies offers unprecedented opportunities to functionally validate and manipulate histone methylation pathways with locus-specific precision. By fusing catalytically inactive dCas9 with histone methyltransferases or demethylases, it is now possible to specifically modulate H3K4me3 or H3K27me3 at individual stress-responsive loci without altering the underlying DNA sequence, thereby enabling the engineering of salt-tolerant crops with minimal risk of off-target genetic changes. The full exploitation of these tools will, however, require a deeper understanding of the cross-talk between histone methylation and other post-translational modifications, including acetylation, phosphorylation, and ubiquitination. The histone code hypothesis posits that it is the combinatorial pattern of marks, rather than any single modification in isolation, that determines chromatin state and transcriptional outcome. Decoding these combinatorial patterns under salinity stress through advanced proteomic and chromatin-mapping approaches will reveal novel molecular targets for crop improvement that remain inaccessible to single-mark analyses.

From an applied perspective, two interconnected priorities deserve particular attention: the mechanistic characterization of transgenerational epigenetic memory and its integration into modern breeding programs. A growing body of evidence supports the hypothesis that histone methylation contributes to stress memory, enabling plants to mount faster and stronger transcriptional responses upon repeated salt exposure, yet fundamental questions regarding the stability, longevity, and heritability of stress-induced methylation marks across generations remain unanswered. Multigenerational epigenomic studies will be required to determine whether stress-induced histone modifications can be stably inherited and whether they can be harnessed to produce crops that are epigenetically primed for improved performance under saline environments. In parallel, the incorporation of epigenetic information into conventional breeding frameworks represents an urgent and underexplored opportunity. Unlike genetic variation, epigenetic variation, including epialleles and differentially methylated regions associated with salinity tolerance, is not captured by current marker-assisted selection platforms. Integrating epigenetic marker data with genomic selection models will improve the predictive accuracy of breeding programs for salt-tolerance traits and accelerate the development of climate-resilient crop varieties.

Finally, the translation of laboratory epigenetic findings into field-applicable crop improvement strategies will require resolution of several practical challenges. The inherently reversible nature of epigenetic modifications raises important questions about their temporal reliability under fluctuating field conditions, and large-scale field trials will be essential to validate the stability and functional effectiveness of epigenetically modified traits across diverse environments. Furthermore, the regulatory landscape governing epigenome-edited crops remains insufficiently defined in many jurisdictions, and clarity in this area will be indispensable for the eventual deployment of CRISPR/dCas9-based epigenetic engineering strategies in commercial agriculture.

## 6. Conclusions

The reviewed evidence demonstrates that histone methylation is a key epigenetic mechanism regulating plant salt-stress tolerance and physiological adaptation. Dynamic changes in active marks such as H3K4me3 and H3K36me3, and repressive marks such as H3K9me2 and H3K27me3, control salt-responsive pathways involved in ion homeostasis, osmotic adjustment, antioxidant defense, hormonal regulation, and stress memory. These processes have direct implications for plant production by supporting growth, reproductive stability, and yield maintenance under saline conditions. From a breeding perspective, salt-responsive histone marks, epialleles, and chromatin-modifying enzymes represent promising biomarkers and targets for developing salinity-tolerant cultivars. Integrating epigenomic information with genomic selection, marker-assisted breeding, and CRISPR/dCas9-based epigenome editing could accelerate the improvement of crops for salt-affected soils. Overall, histone methylation provides both mechanistic insight and practical opportunities for sustainable crop production and breeding under increasing soil salinization.

## Figures and Tables

**Table 1:** Histone methylation marks and their regulatory roles in salt tolerance across plant species.

Crop Species	Histone Mark	Target Genes	Mechanism	Reference
***Arabidopsis thaliana* (L.) Heynh. (Col-0)**	H3K4me3	*RD29A* *RD29B* *COR15A* *COR15B*	H3K4me3 enrichment at stress-responsive gene promoters facilitates transcriptional activation of salinity-induced genes.	[42]
	H3K4me3	*WRKY46* *WRKY70*	JMJ15-mediated H3K4me3 demethylation at *WRKY46* and *WRKY70* represses their expression, enhancing salt tolerance.	[42,53]
	H3K27me3	*HKT1*	Salt stress priming reduces H3K27me3 at *HKT1*, upregulating its expression and limiting shoot Na^+^ accumulation upon recurrent salinity exposure.	[49]
	H3K4me3	*P5CS1*	H3K4me3 retention at *P5CS1* after stress recovery accelerates transcriptional reactivation and proline accumulation under recurrent salinity.	[60]
***Oryza sativa* L.**	H3K4me3 H3K27me3	*OsBZ8*	Cultivar-dependent H3K4me3/H3K27me3 dynamics at *OsBZ8* correlate with differential salt tolerance between Nonabokra and IR64.	[45]
	H3K4me3	*OsHKT1;5*	OsSDG721-mediated H3K4me3 deposition at *OsHKT1;5* upregulates its expression, maintaining K^+^/Na^+^ homeostasis under salinity.	[51]
	H3K27me3 H3K4me3 H3K36me3	No specific targeted gene	Tissue-dependent redistribution of active and repressive histone modifications restructures the stress-responsive transcriptome, with bivalent CS7 states fine-tuning seedling and root salt responses.	[62]
***Medicago sativa* L.**	H3K4me3	*MsMYB4*	H3K4me3 deposition at the *MsMYB4* promoter under salinity drives its transcriptional activation and stress tolerance.	[63]
***Glycine max* (L.) Merr.**	H3K27me3	*Glyma.07G110300Glyma.04G131800Glyma.04G187000Glyma.12G104800*	Salt-induced removal of H3K27me3 at stress-responsive loci alleviates repression and drives their transcriptional activation under salinity.	[64]
	H3K27me3	*Glyma.14G213600Glyma.11G204800Glyma.09G041000Glyma.13G043800Glyma.17G022500*	Salt stress drives de novo H3K27me3 deposition at previously unmarked loci, silencing stress-incompatible genes.	[64]
	H3K9me2	*Glyma11g02400* *Glyma20g30840 Glyma08g41450*	Salinity-driven H3K9me2 depletion relieves repression of MYB, AP2/DREB, and bZIP transcription factors, promoting stress-responsive transcription.	[43]
	H3K4me3 H3K4me2	*GmCHX1 GmCLC1* *SND2*	H3K4 methylation at ion-transport and cell-wall remodeling genes under salt stress upregulates their expression, supporting ionic homeostasis.	[44]
	H3K27me3	*GmZF351*	GmJMJ30-1/2 demethylates H3K27me3 at *GmZF351*, transcriptionally activating it and conferring salt tolerance.	[56]
	H3K4me0/1/2	*GmCYP75B1 GmCYP82C4*	GmPHD6 recognizes H3K4 methylation states at salt-responsive secondary-metabolism loci, regulating their transcriptional output under salinity.	[57]
	H3K4me2 H3K14ac	*GmRD22* *GmGST*	GmPHD5 binding to H3K4me2 recruits GmGNAT1-mediated H3K14 acetylation, activating *GmRD22* and *GmGST* through histone methylation-acetylation cross-talk under salinity.	[65]
***Hordeum vulgare* L.**	H3K4me3 H3K27me3	*HVUL5H46981.2 HVUL1H09481.2*	Co-localised H3K4me3/H3K27me3 at oxidative-defence loci in tolerant cultivar Z0119 enhances peroxidase and catalase (CAT) activity while reducing malondialdehyde (MDA) accumulation under salinity.	[66]
***Ricinus communis* L.**	H3K4me3 H3K27me3	*RSM1*	A salt stress-induced transition from H3K4me3 to H3K27me3 at *RSM1* establishes bivalent chromatin regulation of salt-responsive transcription.	[46]
***Brassica rapa* L.**	H3K27me3	No specific targeted genes	Salinity-induced genome-wide H3K27me3 gain represses hormone signaling and organ development genes, redirecting growth under salt stress.	[67]
***Gossypium hirsutum* L.**	H3K27me3	*GhGSH1* *GhSRM1*	H3K27me3 depletion under KCl stress activates *GhGSH1* and *GhSRM1*, conferring ionic homeostasis and oxidative stress mitigation.	[68]
	H3K27me3	*GhNCED3* *GhTIFY9*	Salt-induced demethylation of H3K27me3 at *GhNCED3* and *GhTIFY9* promotes their expression, reducing oxidative stress and improving ionic homeostasis.	[69]
	H3K4me3	*GhJMJ40*	Salt-responsive *GhJMJ40* encodes a JmjC demethylase inferred to reduce H3K4me3, with yeast complementation confirming a positive contribution to salt tolerance.	[55]
***Zea mays* L.**	H3K36me3	*ZmNHX16 ZmNHX17 ZmHAK24 ZmHAK8 ZmHSP28 ZmUIFG27 ZmIAA9 ZmbHLH32 ZmCAT1* *ZmCLCg*	SDG102-mediated H3K36me3 enrichment activates ion transporter and antioxidant gene networks, collectively improving ionic homeostasis and oxidative stress tolerance.	[70]
	H3K9me2 H3K27me2	No specific targeted gene	Loss of repressive H3K9me2 and H3K27me2 under salinity decondenses chromatin and alters expression at stress-responsive loci.	[71]
	H3K4me2	No specific targeted gene	H3K4me2 enrichment in roots under salinity associates with reduced growth and cell-cycle alteration.	[72]
***Pisum sativum* L.**	H3K4me3 H3K27me3 H3K9ac	*PsPAL* *Ps4CL* *PsCCR* *PsCAD*	Salt-induced H3K4me3 and H3K27me3 enrichment alongside H3K9ac depletion transcriptionally activates phenylpropanoid biosynthesis, with a novel H3K9ac/H3K27me3 bivalent state.	[58]

## Data Availability

No new data were created or analyzed in this study. Data sharing is not applicable to this article.

## References

[1] Raza A., Zaman Q.U., Shabala S., Tester M., Munns R., Hu Z., Varshney R.K. (2025). Genomics-assisted Breeding for Designing Salinity-smart Future Crops. Plant Biotechnol. J..

[2] Melino V., Tester M. (2023). Salt-Tolerant Crops: Time to Deliver. Annu. Rev. Plant Biol..

[3] Sun X., Tan Y., Zhang Y., Guo W., Li X., Golub N., Zhang L., Wang H. (2025). Effects of Salinity Stress on Morphological Structure, Physiology, and MRNA Expression in Different Wheat (*Triticum aestivum* L.) Cultivars. Front. Genet..

[4] Hu Y., Schmidhalter U. (2023). Opportunity and Challenges of Phenotyping Plant Salt Tolerance. Trends Plant Sci..

[5] Hu Y., Wang D., Zhang X., Lv X., Li B. (2025). Current Progress in Deciphering the Molecular Mechanisms Underlying Plant Salt Tolerance. Curr. Opin. Plant Biol..

[6] Peng Y., Zhu H., Wang Y., Kang J., Hu L., Li L., Zhu K., Yan J., Bu X., Wang X. (2025). Revisiting the Role of Light Signaling in Plant Responses to Salt Stress. Hortic. Res..

[7] Balasubramaniam T., Shen G., Esmaeili N., Zhang H. (2023). Plants’ Response Mechanisms to Salinity Stress. Plants.

[8] Talarico E., Greco E., Guarasci F., Araniti F., Chiappetta A., Bruno L. (2025). Epigenetic Regulation of Salt Stress Responses in Rice: Mechanisms and Prospects for Enhancing Tolerance. Epigenomes.

[9] Tibesigwa D.G., Zhuang W., Matola S.H., Zhao H., Li W., Yang L., Ren J., Liu Q., Yang J. (2025). Molecular Insights into Salt Stress Adaptation in Plants. Plant. Cell Environ..

[10] Aufar M., Azwar K. (2026). Morphological and Physiological Responses of Crop Plants to Salinity Stress: A Systematic Review. Contrib. Cent. Res. Inst. Agric..

[11] Hasanuzzaman M., Fujita M. (2022). Plant Responses and Tolerance to Salt Stress: Physiological and Molecular Interventions. Int. J. Mol. Sci..

[12] Liu C., Jiang X., Yuan Z. (2024). Plant Responses and Adaptations to Salt Stress: A Review. Horticulturae.

[13] He X., Zeng J., Cao F., Ahmed I.M., Zhang G., Vincze E., Wu F. (2015). HvEXPB7, a Novel β-Expansin Gene Revealed by the Root Hair Transcriptome of Tibetan Wild Barley, Improves Root Hair Growth under Drought Stress. J. Exp. Bot..

[14] Fu H., Yang Y. (2023). How Plants Tolerate Salt Stress. Curr. Issues Mol. Biol..

[15] Ben Amor N., Jiménez A., Boudabbous M., Sevilla F., Abdelly C. (2020). Chloroplast Implication in the Tolerance to Salinity of the Halophyte Cakile Maritima. Russ. J. Plant Physiol..

[16] Martín-Cardoso H., San Segundo B. (2025). Impact of Nutrient Stress on Plant Disease Resistance. Int. J. Mol. Sci..

[17] Ahmed M.A. (2022). Physiological Effects of Salt Stress on Plant Growth. Tikrit J. Agric. Sci..

[18] Sun M., Liu X., Zheng H., Li L., Lv Q., Wang G. (2025). How Plants Respond to Salt Stress: Lessons Form RBOHs. Plant Sci..

[19] Nigam B., Rathore D., Chaudhary I.J. (2026). Plant Response Mechanisms under Soil Salinity Stress and Its Mitigation and Management Strategies: A Review. J. Plant Biochem. Biotechnol..

[20] Usman M., Wang L., Sun W., An X., Sun Z., Yu F., Liu R., Zhang C. (2026). Integrative Omics and Biotechnological Strategies to Enhance Crop Salinity Tolerance. J. Plant Growth Regul..

[21] Ma L., Xing L., Li Z., Jiang D. (2025). Epigenetic Control of Plant Abiotic Stress Responses. J. Genet. Genom..

[22] Alhammad B.A., Zafar M.M., Alasimi S.M., Seleiman M.F. (2026). Epigenetic Regulation under Light and Temperature Fluctuations: Chromatin Remodeling and Transcriptional Memory in Plants. Plant Cell Rep..

[23] Yang X., Sohail H., Noor I., Costa F.C.L., Zhong S., Zhang L., Chen X. (2025). Epigenetic Crop Improvement: Integrating ENCODE Strategies into Horticultural Breeding. Hortic. Res..

[24] Le H., Simmons C.H., Zhong X. (2025). Functions and Mechanisms of Histone Modifications in Plants. Annu. Rev. Plant Biol..

[25] Qadir M., Kaur N., Rahman F.U., Nabi F., Ahmed Z.F.R., Wu J. (2026). Epigenetic Modifications in Plant Abiotic Stress Adaptation: Towards Climate-Resilient and Sustainable Crop Improvement. Front. Plant Sci..

[26] Keskinoglu M., Sevim G., Gumus B.O., Turkan I., Ozgur R., Uzilday B. (2026). The Interaction between Histone Acetylation and Methylation with ROS Metabolism in Plants. Plant Growth Regul..

[27] Chen C., Li C., Zhu H., Yang J. (2026). Epigenetic Regulation of Salt Stress Responses in Tomato: From DNA Methylation to Stress Memory. Horticulturae.

[28] Yu M.-H., Liao W.-C., Wu K. (2025). Histone Methylation in Plant Responses to Abiotic Stresses. J. Exp. Bot..

[29] Rina S., Xinyue F., Hongmei S. (2026). Dynamic Regulatory Mechanisms of Histone Methylation in Plant Development and Environmental Adaptation. Hortic. Res..

[30] Berger S.L. (2007). The Complex Language of Chromatin Regulation during Transcription. Nature.

[31] Hassan U., Ali Eweda M., Kabir A., Jin X. (2026). Epigenetic Memory and Systemic Priming: An Emerging Framework for Cold-Resilient Crops. Plant Cell Rep..

[32] Xiao M., Wang J., Xu F. (2022). Methylation Hallmarks on the Histone Tail as a Linker of Osmotic Stress and Gene Transcription. Front. Plant Sci..

[33] Sharma M., Sidhu A.K., Samota M.K., Gupta M., Koli P., Choudhary M. (2023). Post-Translational Modifications in Histones and Their Role in Abiotic Stress Tolerance in Plants. Proteomes.

[34] Yung W., Li M., Sze C., Wang Q., Lam H. (2021). Histone Modifications and Chromatin Remodelling in Plants in Response to Salt Stress. Physiol. Plant..

[35] Fang Y., Li J., Jiang J., Geng Y., Wang J., Wang Y. (2017). Physiological and Epigenetic Analyses of Brassica Napus Seed Germination in Response to Salt Stress. Acta Physiol. Plant..

[36] Paes de Melo B., Carpinetti P.d.A., Fraga O.T., Rodrigues-Silva P.L., Fioresi V.S., de Camargos L.F., Ferreira M.F.d.S. (2022). Abiotic Stresses in Plants and Their Markers: A Practice View of Plant Stress Responses and Programmed Cell Death Mechanisms. Plants.

[37] Zhang H., Zhu J., Gong Z., Zhu J.-K. (2022). Abiotic Stress Responses in Plants. Nat. Rev. Genet..

[38] Zhao S., Zhang Q., Liu M., Zhou H., Ma C., Wang P. (2021). Regulation of Plant Responses to Salt Stress. Int. J. Mol. Sci..

[39] Kim J.-M., Sasaki T., Ueda M., Sako K., Seki M. (2015). Chromatin Changes in Response to Drought, Salinity, Heat, and Cold Stresses in Plants. Front. Plant Sci..

[40] Liu Y., Wang J., Liu B., Xu Z. (2022). Dynamic Regulation of DNA Methylation and Histone Modifications in Response to Abiotic Stresses in Plants. J. Integr. Plant Biol..

[41] Singroha G., Kumar S., Gupta O.P., Singh G.P., Sharma P. (2022). Uncovering the Epigenetic Marks Involved in Mediating Salt Stress Tolerance in Plants. Front. Genet..

[42] Shen Y., Chi Y., Lu S., Lu H., Shi L. (2022). Involvement of JMJ15 in the Dynamic Change of Genome-Wide H3K4me3 in Response to Salt Stress. Front. Plant Sci..

[43] Song Y., Ji D., Li S., Wang P., Li Q., Xiang F. (2012). The Dynamic Changes of DNA Methylation and Histone Modifications of Salt Responsive Transcription Factor Genes in Soybean. PLoS ONE.

[44] Yung W., Wang Q., Huang M., Wong F., Liu A., Ng M., Li K., Sze C., Li M., Lam H. (2022). Priming-induced Alterations in Histone Modifications Modulate Transcriptional Responses in Soybean under Salt Stress. Plant J..

[45] Paul A., Dasgupta P., Roy D., Chaudhuri S. (2017). Comparative Analysis of Histone Modifications and DNA Methylation at OsBZ8 Locus under Salinity Stress in IR64 and Nonabokra Rice Varieties. Plant Mol. Biol..

[46] Han B., Xu W., Ahmed N., Yu A., Wang Z., Liu A. (2020). Changes and Associations of Genomic Transcription and Histone Methylation with Salt Stress in Castor Bean. Plant Cell Physiol..

[47] Waters S., Gilliham M., Hrmova M. (2013). Plant High-Affinity Potassium (HKT) Transporters Involved in Salinity Tolerance: Structural Insights to Probe Differences in Ion Selectivity. Int. J. Mol. Sci..

[48] Sun J., Cao H., Cheng J., He X., Sohail H., Niu M., Huang Y., Bie Z. (2018). Pumpkin CmHKT1;1 Controls Shoot Na^+^ Accumulation via Limiting Na^+^ Transport from Rootstock to Scion in Grafted Cucumber. Int. J. Mol. Sci..

[49] Sani E., Herzyk P., Perrella G., Colot V., Amtmann A. (2013). Hyperosmotic Priming of Arabidopsis Seedlings Establishes a Long-Term Somatic Memory Accompanied by Specific Changes of the Epigenome. Genome Biol..

[50] Rus A., Yokoi S., Sharkhuu A., Reddy M., Lee B., Matsumoto T.K., Koiwa H., Zhu J.-K., Bressan R.A., Hasegawa P.M. (2001). AtHKT1 Is a Salt Tolerance Determinant That Controls Na^+^ Entry into Plant Roots. Proc. Natl. Acad. Sci. USA.

[51] Liu Y., Chen X., Xue S., Quan T., Cui D., Han L., Cong W., Li M., Yun D., Liu B. (2021). SET DOMAIN GROUP 721 Protein Functions in Saline–Alkaline Stress Tolerance in the Model Rice Variety Kitaake. Plant Biotechnol. J..

[52] Jeevaraj T., Blessy A., Krishnamoorthy S., Sridhar A. (2025). Histone Demethylation by JMJ Family Genes: Insights into Plant Growth and Adaptation. Plant Mol. Biol..

[53] Shen Y., Conde e Silva N., Audonnet L., Servet C., Wei W., Zhou D.-X. (2014). Over-Expression of Histone H3K4 Demethylase Gene *JMJ15* Enhances Salt Tolerance in *Arabidopsis*. Front. Plant Sci..

[54] Chowrasia S., Panda A.K., Rawal H.C., Kaur H., Mondal T.K. (2018). Identification of JumonjiC Domain Containing Gene Family among the *Oryza* Species and Their Expression Analysis in FL478, a Salt Tolerant Rice Genotype. Plant Physiol. Biochem..

[55] Sun Z., Wang X., Qiao K., Fan S., Ma Q. (2021). Genome-Wide Analysis of JMJ-C Histone Demethylase Family Involved in Salt-Tolerance in *Gossypium hirsutum* L.. Plant Physiol. Biochem..

[56] Wei W., Lu L., Bian X., Li Q., Han J., Tao J., Yin C., Lai Y., Li W., Bi Y. (2023). Zinc-finger Protein GmZF351 Improves Both Salt and Drought Stress Tolerance in Soybean. J. Integr. Plant Biol..

[57] Wei W., Tao J.-J., Chen H.-W., Li Q.-T., Zhang W.-K., Ma B., Lin Q., Zhang J.-S., Chen S.-Y. (2017). A Histone Code Reader and a Transcriptional Activator Interact to Regulate Genes for Salt Tolerance. Plant Physiol..

[58] Wan H., Cao L., Wang P., Hu H., Guo R., Chen J., Zhao H., Zeng C., Liu X. (2024). Genome-Wide Mapping of Main Histone Modifications and Coordination Regulation of Metabolic Genes under Salt Stress in Pea (*Pisum sativum* L.). Hortic. Res..

[59] Perrella G., Fasano C., Donald N.A., Daddiego L., Fang W., Martignago D., Carr C., Conti L., Herzyk P., Amtmann A. (2024). Histone Deacetylase Complex 1 and Histone 1 Epigenetically Moderate Stress Responsiveness of *Arabidopsis thaliana* Seedlings. New Phytol..

[60] Feng X.J., Li J.R., Qi S.L., Lin Q.F., Jin J.B., Hua X.J. (2016). Light Affects Salt Stress-Induced Transcriptional Memory of *P5CS1* in *Arabidopsis*. Proc. Natl. Acad. Sci. USA.

[61] Fu Y.-L., Zhang G.-B., Lv X.-F., Guan Y., Yi H.-Y., Gong J.-M. (2013). *Arabidopsis* Histone Methylase CAU1/PRMT5/SKB1 Acts as an Epigenetic Suppressor of the Calcium Signaling Gene *CAS* to Mediate Stomatal Closure in Response to Extracellular Calcium. Plant Cell.

[62] Zheng D., Wang L., Chen L., Pan X., Lin K., Fang Y., Wang X., Zhang W. (2019). Salt-Responsive Genes Are Differentially Regulated at the Chromatin Levels between Seedlings and Roots in Rice. Plant Cell Physiol..

[63] Dong W., Gao T., Wang Q., Chen J., Lv J., Song Y. (2020). Salinity Stress Induces Epigenetic Alterations to the Promoter of MsMYB4 Encoding a Salt-Induced MYB Transcription Factor. Plant Physiol. Biochem..

[64] Sun L., Song G., Guo W., Wang W., Zhao H., Gao T., Lv Q., Yang X., Xu F., Dong Y. (2019). Dynamic Changes in Genome-Wide Histone3 Lysine27 Trimethylation and Gene Expression of Soybean Roots in Response to Salt Stress. Front. Plant Sci..

[65] Wu T., Pi E.-X., Tsai S.-N., Lam H.-M., Sun S.-M., Kwan Y.W., Ngai S.-M. (2011). GmPHD5 Acts as an Important Regulator for Crosstalk between Histone H3K4 Di-Methylation and H3K14 Acetylation in Response to Salinity Stress in Soybean. BMC Plant Biol..

[66] Yuzhen B., Sang Z., Mu W., Yu M., Wang Y., Yuan H., Xu Q. (2021). Whole-Genome Analysis of the Trimethylation of Histone H3 Lysine 4 and Lysine 27 in Two Contrasting Tibetan Hulless Barley Genotypes under Salinity Stress. Acta Physiol. Plant..

[67] Liang H., Wang Q., He H., Zhang X., Wang Z., Zhi Y., Zhang B., Ma W., Liu Z., Liu F. (2026). The Genome-Wide Landscape of Histone Modifications Dynamics in Non-Heading Chinese Cabbage Root Tips under Salt Stress. J. Integr. Agric..

[68] Zhang Z., Huo W., Li X., Ren Z., Liu Y., He K., Zhang F., Guo J., Ma X., Yang D. (2026). Histone H3K27 Trimethylation Modulates the Cotton Response to KCl-Induced Ionic Stress. Plant Physiol..

[69] Liu W., Zhang Z., Du Q., Li J., Yang L., Ren Z., Guo J., Zhu W., Ma Z., Zhou Y. (2026). H3K27me3-Mediated Chromatin Remodeling Governs Salt Tolerance in Cotton via Na^+^/K^+^ Homeostasis Modulation. J. Adv. Res..

[70] Liu X., Zheng Y., Liu Y., Wang H., Wei X., Liu H., Tian H., Zhao X., Wang Z., Qi X. (2026). H3K36 Methyltransferase SDG102 Enhances Salt Tolerance by Altering the Methylation Level of Genes in Maize (*Zea mays* L.). BMC Plant Biol..

[71] Kamal K.Y., Khodaeiaminjan M., Yahya G., El-Tantawy A.A., Abdel El-Moneim D., El-Esawi M.A., Abd-Elaziz M.A.A., Nassrallah A.A. (2021). Modulation of Cell Cycle Progression and Chromatin Dynamic as Tolerance Mechanisms to Salinity and Drought Stress in Maize. Physiol. Plant..

[72] Zhao L., Wang P., Hou H., Zhang H., Wang Y., Yan S., Huang Y., Li H., Tan J., Hu A. (2014). Transcriptional Regulation of Cell Cycle Genes in Response to Abiotic Stresses Correlates with Dynamic Changes in Histone Modifications in Maize. PLoS ONE.

[73] Hemenway E.A., Gehring M. (2023). Epigenetic Regulation during Plant Development and the Capacity for Epigenetic Memory. Annu. Rev. Plant Biol..

[74] Gallusci P., Agius D.R., Moschou P.N., Dobránszki J., Kaiserli E., Martinelli F. (2023). Deep inside the Epigenetic Memories of Stressed Plants. Trends Plant Sci..

[75] Liu J., He Z. (2020). Small DNA Methylation, Big Player in Plant Abiotic Stress Responses and Memory. Front. Plant Sci..

[76] Crisp P.A., Ganguly D., Eichten S.R., Borevitz J.O., Pogson B.J. (2016). Reconsidering Plant Memory: Intersections between Stress Recovery, RNA Turnover, and Epigenetics. Sci. Adv..

[77] Ganie S.A., McMulkin N., Devoto A. (2024). The Role of Priming and Memory in Rice Environmental Stress Adaptation: Current Knowledge and Perspectives. Plant. Cell Environ..

[78] Zhang D., Zhang D., Zhang Y., Li G., Sun D., Zhou B., Li J. (2024). Insights into the Epigenetic Basis of Plant Salt Tolerance. Int. J. Mol. Sci..

[79] Siddique A.B., Parveen S., Rahman M.Z., Rahman J. (2024). Revisiting Plant Stress Memory: Mechanisms and Contribution to Stress Adaptation. Physiol. Mol. Biol. Plants.

[80] Karalija E., Ibragić S., Dahija S., Šamec D. (2025). Transgenerational Memory of Phenotypic Traits in Plants: Epigenetic Regulation of Growth, Hormonal Balance, and Stress Adaptation. Curr. Issues Mol. Biol..

[81] Turgut Kara N., Arıkan B., Pulat E., Çakır Ö. (2025). The Role of Plant Epigenetic Memory Mechanisms in Abiotic Stress Tolerance. Epigenetic for Climate-Smart and Sustainable Agriculture.

[82] Zulfiqar F., Ashraf M. (2023). Proline Alleviates Abiotic Stress Induced Oxidative Stress in Plants. J. Plant Growth Regul..

[83] Arıkan B., Çakır Ö., Kara N.T. (2025). Epigenetic and Transcriptomic Features of Stress Memory during Salt-Stress Priming in Arabidopsis. Plant Sci..

[84] Verma N., Dhall R.K., Yadav S., Rana N., Kaur M., Kumari P., Kumar P., Maheshwari R., Sharma P. (2026). Genetic and Molecular Breeding Perspectives on Developing Abiotic Stress Tolerant Pea (*Pisum sativum* L.). Front. Plant Sci..

[85] Shi L., Cui X., Shen Y. (2024). The Roles of Histone Methylation in the Regulation of Abiotic Stress Responses in Plants. Plant Stress.

[86] Guo P., Chong L., Jiao Z., Xu R., Niu Q., Zhu Y. (2025). Salt Stress Activates the CDK8-AHL10-SUVH2/9 Module to Dynamically Regulate Salt Tolerance in *Arabidopsis*. Nat. Commun..

[87] Sun Y., Zhao J., Li X., Li Y. (2020). E2 Conjugases UBC1 and UBC2 Regulate MYB42-mediated SOS Pathway in Response to Salt Stress in Arabidopsis. New Phytol..

[88] Nguyen N.H., Jung C., Cheong J.-J. (2019). Chromatin Remodeling for the Transcription of Type 2C Protein Phosphatase Genes in Response to Salt Stress. Plant Physiol. Biochem..

[89] Cheng X., Zhang S., Tao W., Zhang X., Liu J., Sun J., Zhang H., Pu L., Huang R., Chen T. (2018). INDETERMINATE SPIKELET1 Recruits Histone Deacetylase and a Transcriptional Repression Complex to Regulate Rice Salt Tolerance. Plant Physiol..

[90] Yin W., Xiao Y., Niu M., Meng W., Li L., Zhang X., Liu D., Zhang G., Qian Y., Sun Z. (2020). ARGONAUTE2 Enhances Grain Length and Salt Tolerance by Activating BIG GRAIN3 to Modulate Cytokinin Distribution in Rice. Plant Cell.

[91] Wang S., Jiang L., Zhai T., Qu K., Liu X., Di Z., Chen Y., Lu X., Li X., Zhang J. (2025). The JmjC Domain-Containing Histone Demethylase ZmJMJ703 Orchestrates Salt Stress Adaptation in Maize. J. Plant Physiol..

